# Acute respiratory distress syndrome among patients with severe COVID-19 admitted to treatment center of Wollega University Referral Hospital, Western Ethiopia

**DOI:** 10.1371/journal.pone.0267835

**Published:** 2022-06-16

**Authors:** Tadesse Tolossa, Emiru Merdassa Atomssa, Getahun Fetensa, Lami Bayisa, Diriba Ayala, Ebisa Turi, Bizuneh Wakuma, Diriba Mulisa, Dejene Seyoum, Ayantu Getahun, Tesfaye Shibiru, Ginenus Fekadu, Markos Desalegn, Haile Bikila

**Affiliations:** 1 Department of Public Health, Institute of Health Sciences, Wollega University, Nekemte, Ethiopia; 2 Department of Nursing, Institute of Health Sciences, Wollega University, Nekemte, Ethiopia; 3 Department of Hea lth Behavior and Society, Institute of Health, Jimma University, Jimma, Ethiopia; 4 Department of Midwifery, Institute of Health Sciences, Wollega University, Nekemte, Ethiopia; 5 School of Medicine, Institute of Health Sciences, Wollega University, Nekemte, Ethiopia; 6 Department of Pharmacy, Institute of Health Sciences, Wollega University, Nekemte, Ethiopia; 7 School of Pharmacy, Faculty of Medicine, The Chinese University of Hong Kong, Shatin, New Territories, Hong Kong; Gulu University, UGANDA

## Abstract

**Background:**

Acute respiratory distress syndrome is a life-threatening condition that has a significant effect on the occurrence of morbidity and mortality among patients with severe Coronavirus disease 2019 (COVID-19). To the best of researchers’ knowledge, there is no Study on ARDS of COVID-19 in Ethiopia. Therefore, this study aimed to identify the prevalence of ARDS and associated factors among severe COVID-19 patients at Wollega University Referral Hospital.

**Methods:**

An institution-based retrospective cross-sectional study was conducted from September 20, 2020, to June 10, 2021. Real-Time Reverse transcription-polymerase Chain Reaction (rRT-PCR) test was used to test Patients for COVID-19. Epi-data version 3.2 was used for data entry, and the final data analysis was through STATA version 14. After checking the assumption P-value<0.25 in the bivariable analysis was used to select a candidate variable for multi-variable analysis, and a p-value of <0.05 was used to declare statistical significance.

**Results:**

In this study, the prevalence of ARDS was 32%. Almost all the patients had the clinical feature of cough (93.7%), followed by shortness of breath (79.9%), fever (77.7%), and headache (67%). Age older than 65 years (AOR = 3.35, 95%CI = 1.31, 8.55), male gender (AOR = 5.63, 95%CI = 2.15, 14.77), and low oxygen saturation level (AOR = 4.60, 95%CI = 1.15, 18.35) were the independent predictors of ARDS among severe COVID-19 patients.

**Conclusion:**

The prevalence of ARDS among patients with severe COVID-19 was high in the study area. Therefore, elders and patients with critical conditions (low oxygen saturation) better to get special attention during COVID-19 case management to enhance good care and monitoring of the patients.

## Introduction

Acute respiratory distress syndrome is a rapidly progressive disorder that initially manifests as shortness of breath, tachypnea, and low oxygen saturation, which quickly lead to respiratory failure [1]. ARDS is a life-threatening condition, a potentially fatal respiratory condition in which the lungs are severely swelling and inflamed, causing small blood vessels to leak fluid and the collapse of alveoli due to the filling of fluid, preventing the proper functioning of the lungs and finally affecting the ability to give the organs with enough oxygen [2]. Patients with ARDS must be given extra oxygen to maintain enough oxygen in the blood, which usually needs a ventilator to breathe. The ARDS might be caused by pneumonia, aspiration (inhalation of stomach contents into the lungs), inhalation of toxic substances, bruising of the lungs caused by chest trauma, near-drowning, fat embolism (when a clot of fat enters the pulmonary circulation), lung transplantation, a viral infection of the lungs, including the coronavirus that causes COVID-19 infection [3–5].

Coronavirus disease-19 (COVID-19) is one of the world’s major public health problems. As of July 19, 2021, globally this disease had affected 222 countries with a total of 190,752,362 cases and 4,093,268 deaths. Ethiopia had a total cases of 277,696 and total death of 4,357 [6]. COVID-19 has several severe forms, of which acute respiratory distress syndrome [4] is the common one [7, 8]. But, ARDS caused by COVID-19 is considerably different from ARDS caused by other factors. Even, the onset time of COVID- 19 associated ARDS is 8 to 12 days, based on Berlin ARDS criteria, the onset limit is 1 week [1, 9].

ARDS complications can lead to blood clots, collapsed lungs, infections, pulmonary fibrosis, and organ damage or failure. Apart from these, patients recovery from COVID-19 results in potentially lasting effects like depression, breathing problems, memory and thinking problems, and muscle weakness which may persist for years [2, 10].

A comparative study conducted in the United States reveals that the Prevalence of ARDS was two times as large as before COVID-19 [11]. This can be supplemented by other evidence from western New Yorka, 29.4% of them were diagnosed with ARDS [12]. Furthermore, 56.1% and 22.3% of COVID 19 patients were presented with ARDS in Madrid, Spain and Juba, South Sudan [13], respectively. On top to this, a study done in Spain indicated the prevalence of ARDS among admitted COVID-19 cases was 31.5% [14]. Literatures showed that old age, diabetes, smoking cigarettes, alcohol use, having chronic liver disease, immune-compromised, and being hypertensive were significant predictors of ARDS among severe COVID-19 patients [15, 16].

ARDS has a significant effect on the occurrence of morbidity and mortality among severe COVID-19 patients. According to studies conducted across different parts of the world, over 40% of death was attributed to acute respiratory syndrome [16–18]. A study conducted in China shows, the rate of mortality due to ARDS among COVID-19 cases was 39% [18]. The other pocket study conducted in a Polish national hospital shows the magnitude of mortality due to COVID-19 associated ARDS was 88.8% [19]. In addition, the health facilities are overburdened and increased utilization of critical care services by COVID-19 patients. According to a study conducted in 50 selected countries, the ARDS accounts for 10.4% of intensive care unit (ICU) admissions, and 23.4% of ICU patients that require mechanical ventilation [20]. Patients with ARDS have a large financial cost because of longer hospitalization with serious physical and emotional consequences for those recovering from very severe COVID-19 [21].

In Ethiopia, the Prevalence of ARDS among severe COVID-19 patients is unknown. Therefore, this study aimed to identify the prevalence of acute respiratory distress syndrome and associated factors among severe COVID-19 patients at Wollega University Referral Hospital.

## Methods

### Study setting and design

An institution-based retrospective cross-sectional study was conducted at Wollega University referral hospital COVID-19 treatment center, which is found in Western Ethiopia at a distance of 327 Kilo meters to the west of Addis Ababa, the capital city of the country. The hospital was the first treatment center in the Western part of the country in the early phase of the pandemic in Ethiopia. The data were retrieved between June 15–25, 2021from all records of patients admitted with COVID-19between September 20, 2020 to June 10, 2021.

### Source population

All records of patients who tested positive for COVID-19 by using the Real-Time Reverse transcription-polymerase Chain Reaction (rRT-PCR) test and admitted to the WURH treatment center during the study period were included.

### Study population

All sampled records of patients who tested positive for COVID-19 by using the rRT-PCR test and admitted to the WURH treatment center during the study period were included.

### Eligibility criteria

#### Inclusion criteria

All records of COVID-19 Patients admitted to WURH from September 20, 2020, to June 10, 2021, were included in the study.

#### Exclusion criteria

Records of COVID-19 patients lacking outcome variables were excluded.

#### Sample size and sampling techniques

All Covid-19 patients admitted to the treatment center during the study period with the complete medical records were included in this study. A total of 417 patients were admitted with severeCOVID-19 cases to the WURH treatment center, of which 318 severe COVID-19 cases with complete data were included in this study.

#### Variables and measurement of outcome

Prevalence of respiratory distress syndrome [4] was the outcome variable for this study. ARDS was diagnosed clinically and radiologic investigation by the Physician from the patient on admission. Clinically, ARDS was diagnosed when the patient reports two or more clinical manifestations such as cough, fever, sore throat, and shortness of breath [22], whereas, Radiologic definition of ARDS was defined according to Kigali modification which is the presence of bilateral opacities at chest radiograph or lung ultrasound and hypoxia with a cutoff of SpO2/FIO2 less than or equal to 315 [1].

Comorbidity (Yes/No) was the co-existence of one or more diseases with severe COVID-19 cases “Yes” and, if not it was considered as “No”. Random blood sugar (RBS) was categorized as normal and above the normal range [23]. A normal level of RBS was a level of less than or equal to 200 mg/dl, and RBS greater than 200 mg/dl was an indication of diabetes. Oxygen saturation was categorized as normal oxygen levels in a pulse oximeter which is range from 94% to 100%, and blood oxygen levels below 94% are considered low (hypoxemia).

#### Data collection tools and procedure

Data were collected from the medical cards of the patients. The data extraction tool was prepared by reviewing patient medical cards and similar kinds of literature conducted in this area. The checklist consists of, **Section I**: three variables as socio-demographic related variables (age, sex, and residence of participants); **Section II** consists of 14 variables as clinical (fever on admission, headache on admission, pain on admission, loss of appetite, sore throat, cough on admission, SOB on admission, fatigue, status of the patients on admission, organ failure, oxygen supplemented, activity, duration of clinical manifestation on arrival to treatment center, comorbidity on admission); **Section III** consists of types of medication and laboratories results. During data collection time, the outcome was confirmed by reviewing the chart, which was recorded by the physician. The one-day training was given for data collectors by the principal investigator. Trained health professionals who have been working in the treatment center were recruited for data extraction. A pre-test was conducted on 5% of patient records before the actual data collection to check the clarity of the checklist, and availability of variables on the card of the patients. All data collectors and supervisors used personal protective equipment’s as per standard for COVID-19 caregivers during data collection.

#### Data management and analysis

Epi-data version 3.2 was used for data entry, and the data were exported to STATA version 14 for further analysis. Before analysis, the data were cleaned, and edited using simple frequencies and cross-tabulation; re-categorization of categorical variables and categorization of continuous variables was done to be suitable for analysis. Descriptive statistics, like frequencies, percentages, mean and standard deviation were computed. A logistic regression model was fitted to determine factors associated with respiratory distress. The assumption of the logistic regression model was checked by the chi-square test. Factors associated with respiratory distress were screened at a p-value < 0.25 in bi-variable analysis to be candidates for multivariable analysis. Adjusted Odds Ratios (AOR) with 95% confidence intervals was computed and statistical significance was declared when it is significant at the 5% level (p-value< 0.05) in the final model.

Multicollinearity was checked using the variance inflation factor (VIF) and all the covariates had a VIF value of less than 10, confirming that there is no indication of severe Multicollinearity (Table 1).

**Table 1:** Multicollinearity assessment output.

Variables	VIF
Oxygen saturation level	2.88
Gender	2.43
Neutrophilia	1.61
Age group	1.29
Mean VIF	1.86

#### Ethical consideration

Ethical clearance was obtained from Wollega University Research Review Committee (WURRC). A formal letter of cooperation was written to the WURH treatment center and permission was obtained from the hospital administration. Informed consent was obtained from all the participants or responsible third-party caregivers. Personal identifiers were not used on the data collection checklist. To ensure confidentiality, the name and other identifiers of participants and health care professionals were not recorded on the data collection tools. All methods were performed by in relevant guidelines and regulations.

## Results

Out of 417 patients with severe COVID-19 admitted to the WURH treatment center, 99 patient cards were excluded from analysis due to unregistered outcomes and incomplete data.

### Socio-demographic characteristics and findings of the patients

Most participants were female (67.92%), urban dwellers (58.49%), and more than half (55.66%) had no baseline comorbidity. The majority of the patients had encountered fever (74.21%) and shortness of breath (80.50%). On admission, more than half (55.35%) of the patients were recorded to have subcritical status. More than two-thirds of the patients were supplemented with Oxygen (70.13%), having Oxygen saturation <94(73.58%). As to the chest x-ray finding, nearly one-third (32.1%) of the patients had bilateral lung infiltration, and had neutrophilia (44.34%) as reported by the physician. Most of the patients (90.57%) were delayed more than 24hrs to get admission and among admitted patients (44.34%) were stayed in the hospital for more than eleven days (Table 2).

**Table 2:** Socio-demographic characteristics and findings of patients admitted with severe COVID-19 at WURH, 2021.

Variables	Variables	Frequency (n)	Percent (%)
Age (years)	<65	274	86.16
	≥65	44	13.84
Gender	Male	102	32.08
	Female	216	67.92
Residence	Rural	132	41.51
	Urban	186	58.49
Comorbidity	No	177	55.66
	Yes	141	44.34
Comorbidity	Hypertension	68	48.23
	Diabetes Mellitus	41	29.08
	Chronic Heart Failure	22	15.60
	Stroke	1	0.71
	Tuberculosis	14	9.93
	HIV/AIDS	15	10.64
	Asthma	17	12.06
	Chronic Kidney Disease	4	2.84
	Others (Anemia, Dyspepsia, and Epilepsy	10	7.09
Status of patients	Stable	58	18.24
	Subcritical	176	55.35
	Critical	84	26.42
Oxygen supplemented	No	95	29.87
	Yes	223	70.13
Oxygen saturation levels (%)	≥94	84	26.42
	<94	234	73.58
Received intranasal oxygen	No	21	6.60
	Yes	297	93.40
Types of oxygen	Face mask	79	26.60
	Intranasal	218	73.40
Chest X-ray results	no infiltrates	126	39.6
	Bilateral	102	32.1
	Unilateral	90	28.3
Neutrophilia	No (≤7.7×10^9/L)	177	55.66
	Yes (>7.7×10^9/L)	141	44.34
Duration of hospital stay	≤11days	177	55.66
	>11days	141	44.34
Time from onset to admission	Early (within 48hrs)	30	9.43
	Delayed (more than 48hrs)	288	90.57

In this study, numbers of encountered baseline comorbidities were identified among patients admitted with severe COVID-19.

### Common signs and symptoms reported

**Fig 1:**
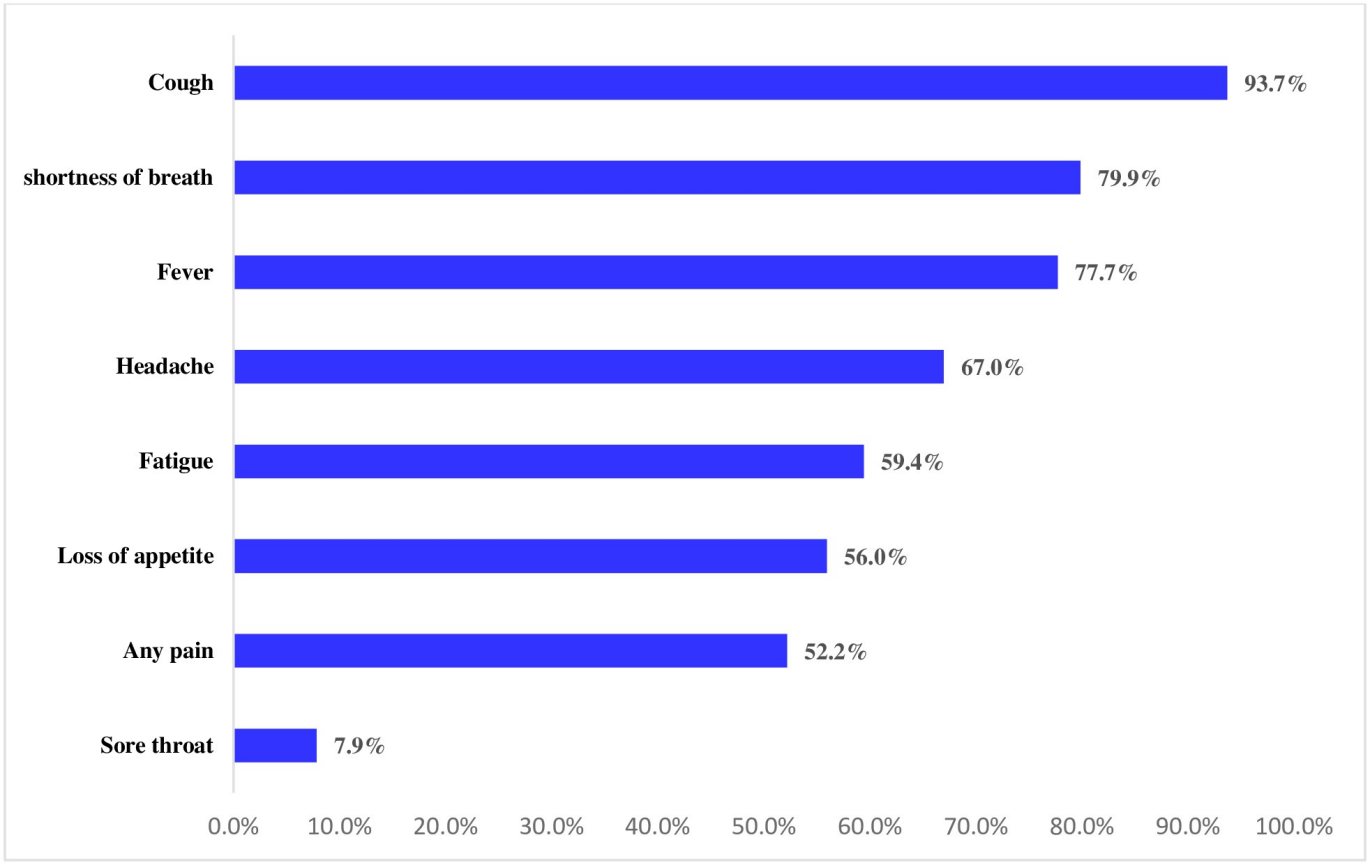
Sign and symptoms reported among severe COVID-19 patients admitted at WURH, 2021.

Regarding the sign and symptoms that were reported among the patient, almost all of the patients had the clinical features of cough (93.7%), followed by shortness of breath (79.9%), fever (77.7%), and headache (67%) whereas the fewer sign/symptom patients reported was sore throat (7.9%) (Fig 1).

### List of medications prescribed for patients

**Fig 2:**
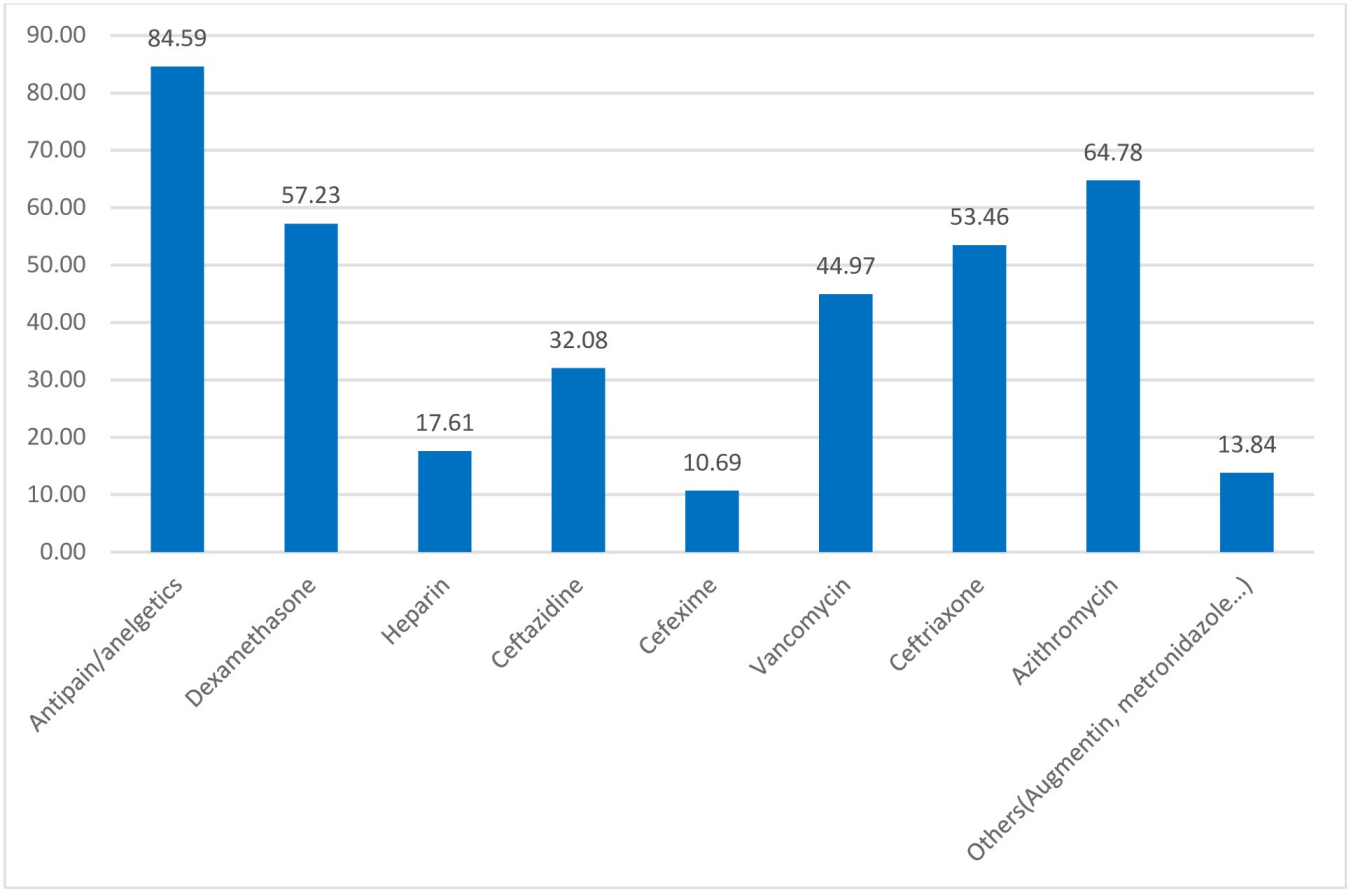
Percentage distribution of types of drugs used for treating severe COVID-19 patients admitted at WURH, 2021.

Different drugs from different class categories have been used for the treatment of patients. Accordingly, about five in six patients (84.59%) were treated with ant pain/analgesics followed by about two-third (64.78%) were treated with azithromycin. More than half were treated with dexamethasone (57.23%), ceftriaxone (53.46%), and two-fifth (44.97%) were treated with Vancomycin (Fig 2).

### Prevalence of acute respiratory distress syndrome

**Fig 3:**
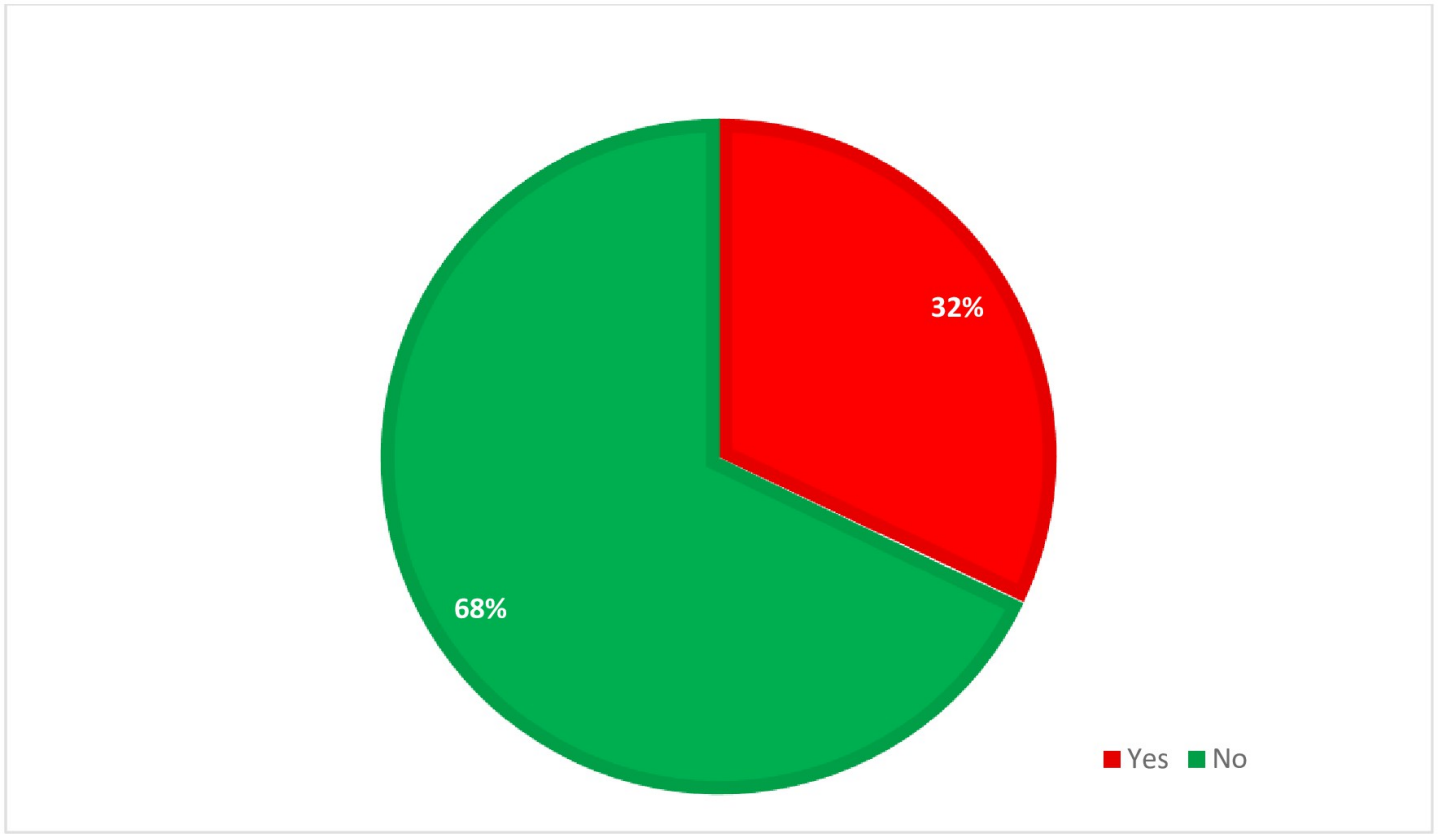
Magnitude of acute respiratory distress syndrome reported among severe COVID-19 patients included in the study at WURH, 2021.

Among a total of 318 patients who were admitted with severe covid-19 and included in the study, **32%** of patients were reported with ARDS (Fig 3).

### Bivariate and multivariate analysis of ARDS

Bivariate analysis was conducted. The association between acute respiratory distress syndrome and gender, age group, comorbidity, oxygen saturation level, neutrophilia, and duration of hospital stay has P<0.25 for each variable. These variables were included in the multivariate analysis. The results from multivariable logistic regression analysis showed that the factors associated with acute respiratory distress syndrome were age group, gender, and oxygen saturation levels (Table 3).

**Table 3:** Bivariate analysis for acute respiratory distress syndrome among patients admitted with severe COVID-19 at WURH, 2021.

Variables	Category	ARDS				χ^2^	P-value
		No		Yes			
		N	%	N	%		
Age (years)	<65	191	69.71	83	30.29	2.89	0.089*
	≥65	25	56.82	19	43.18		
Gender	Female	74	72.55	28	27.45	1.47	0.225
	Male	142	65.74	74	34.26		
Residence	Rural	92	69.70	40	30.30	0.32	0.568
	Urban	124	66.67	62	33.33		
Comorbidity	No	126	71.19	51	28.81	1.95	0.163*
	Yes	90	63.83	51	36.17		
Fever	No	56	68.29	26	31.71	0.07	0.934
	Yes	160	67.80	76	32.20		
O_2_ saturation levels (%)	≥94%	64	76.19	20	23.81	3.58	0.058*
	<94%	152	64.96	82	35.04		
Neutrophilia	No	152	71.03	62	28.97	2.89	0.089*
	Yes	64	61.54	40	38.46		
Duration of hospital stay	≤11days	129	72.88	48	27.12	4.50	0.034*
	>11days	87	61.70	54	38.30		
Time from onset to administration	Early	21	70.00	9	30.00	0.07	0.798
	Delayed	195	67.71	93	32.29		

**Fig 4:**
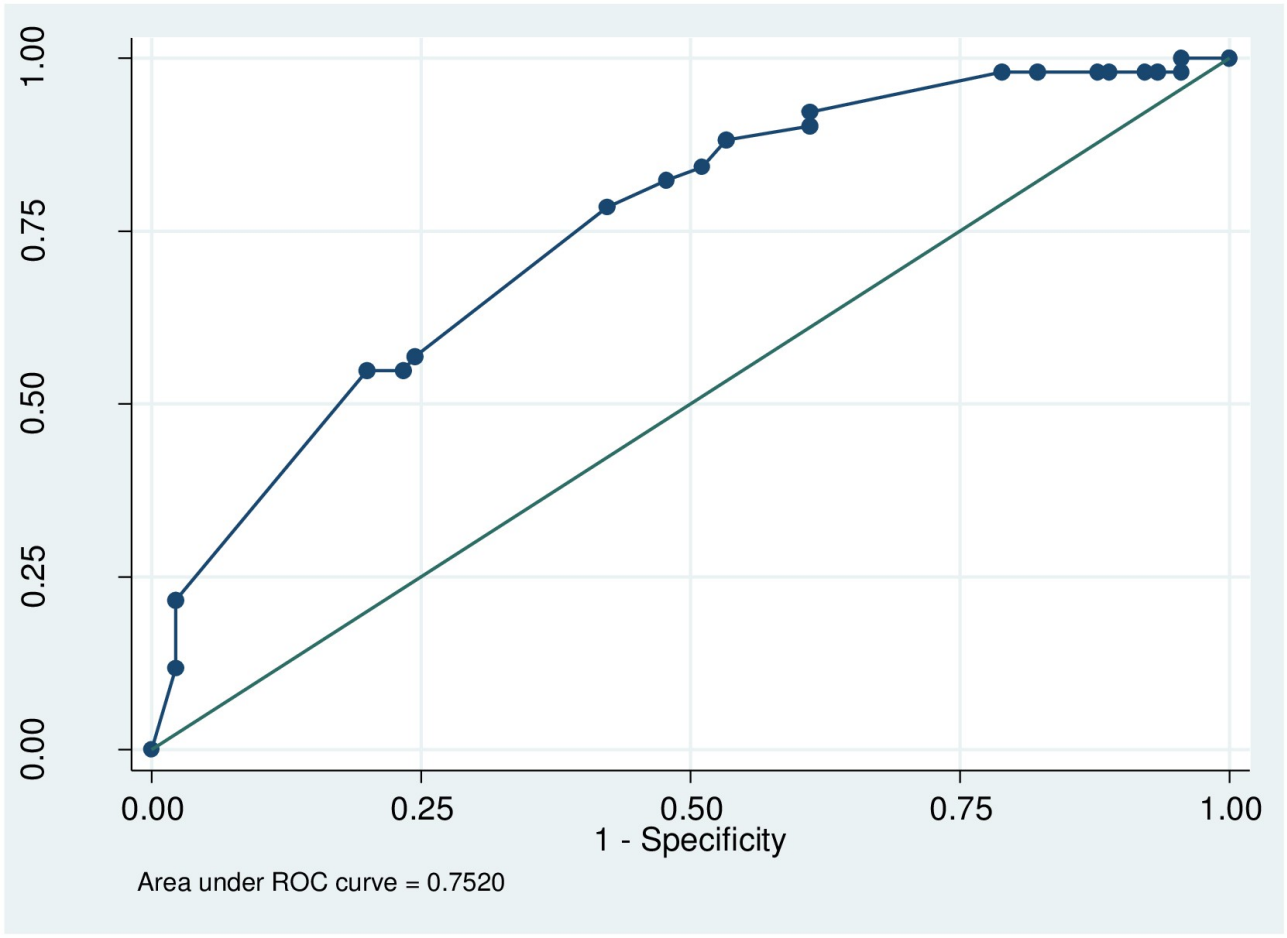
Model goodness of fit. $\begin{array}{cc} \text{number}\;\text{of}\;\text{observations}= & 141\\ \text{number}\;\text{of}\;\text{groups}= & \;7\\ \text{Hosmer-Lemeshow}\;\text{chi}2\left(5\right)= & \;0.95\\ \text{Prob}>\text{chi}2= & \;0.9667 \end{array}$.

Final model was fitted the data, Hosmer-Lemshowchi-squared = 0.95, p value = 0.9667 (Fig 4).

Compared to the patients aged younger than 65 years, the patients older than 65 were about 3 times more likely to have acute respiratory distress syndrome (OR = 3.35, 95%CI = 1.31, 8.55). The odds of acute respiratory distress syndrome were more than fivefold more likely for males compared to the female (AOR = 5.63, 95%CI = 2.15, 14.77). The patient’s oxygen saturation level was one of the variables that have associated with acute respiratory distress syndrome. Patient with low oxygen concentration levels has 4.6 times more likely to have acute respiratory distress syndrome (AOR = 4.60, 95%CI = 1.15, 18.35). The other variables residence, existence of comorbidity, neutrophilia, duration of hospital stays, and time from onset to admission were not found to be statistically significantly associated with the acute respiratory distress syndrome of these severe COVID-19 patients (Table 4).

**Table 4:** Multivariable analysis for acute respiratory distress syndrome among patients admitted with severe COVID-19 at WURH, 2021.

Variables	Category	ARDS		COR, 95%CI	AOR, 95%CI
		No	Yes		
Age (years)	<65	191	83	Ref	Ref
	≥65	25	19	1.75(0.91, 3.35)	3.35 (1.31, 8.55) *
Gender	Female	74	28	Ref	Ref
	Male	142	74	1.38(0.82, 2.31)	5.63 (2.15, 14.77) *
Comorbidity	No	126	51	Ref	Ref
	Yes	90	51	1.40(0.87, 2.25)	1.23(0.75, 2.01)
O_2_ saturation levels (%)	≥94%	64	20	Ref	Ref
	<94%	152	82	1.73(0.97, 3.05)	4.60 (1.15, 18.35) *
Neutrophilia	No	152	62	Ref	Ref
	Yes	64	40	1.53(0.94, 2.51)	0.65(0.29, 1.45)
Duration of hospitalstay	≤11days	129	48	Ref	Ref
	>11days	87	54	1.67(1.04, 2.68)	1.66(0.76, 3.64)
Time from onset to admission	Early	21	9	Ref	Ref
	Delayed	195	93	1.11(0.49, 2.52)	0.81(0.16, 3.96)

*Statistically significance at p-value <0.05

## Discussion

ARDS is an acute systematic inflammatory response, which can be caused by insult to the lung either direct or indirect. In severe cases, COVID-19 can be complicated by acute respiratory distress syndrome [24]. Injury to the alveolar epithelial cells is the main cause of COVID-19-related ARDS [25]. Thus, this study was conducted to assess the Prevalence and factors associated with ARDS among severe COVID-19 patients admitted to WURH.

In this study, nearly one-third (32%) of the study subjects had ARDS. This figure was lower than the study findings from a study conducted in China and the USA which depicted the prevalence of ARDS was 41.8% [26] and 67% [27], respectively. The possible reason for the discrepancy might be, the previous studies were conducted in more industrialized settings where respiratory problems are prevalent. Also, the previous studies conducted among COVID-19 admitted to ICU in which patients who has been on mechanical ventilation for several days are potentially at risk of developing pulmonary fibrosis leading to potential restrictive to lung disease and increases the prevalence of ARDS.

However, this finding was higher than study result conducted previously that reports the P prevalence of ARDS as of 11.5% [28]. The difference might be due to the previous study was conducted at multicenter setting to compare COVID-19 ARDS with non-COVID-19 ARDS but in case of current study, the study was conducted at a single facility on admitted patients with severe COVID-19 that could increases the Prevalence of ARDS.

ARDS is one of the complications manifested in severe COVID-19 patients and it is also responsible for extra transience. Odds for developing ARDS were 3-fold higher in subjects with the age ≥65 years old as compared to those with the age <65 years old. Similar finding were reported from different setting [26, 28, 29], the risk of severe illness with COVID-19 increases with age, older adults are more likely to be hospitalized or die. Possible reason, older people may have pre-existing other medical conditions such as asthma and chronic obstructive pulmonary disease (COPD) to be more vulnerable to becoming develop ARDS and potential physiological changes that come with ageing may put them at significant risk of developing severe illness. As well, the odds of developing ARDS were 6-folds higher in male patients when compared to female individuals. This result is supported by previous study findings [28, 30, 31], in which male patients were reported as more likely to develop ARDS. Possible reason: Generally, females are more resistant to infection than men, and this is possibly mediated by several factors including sex hormones and high expression of coronavirus receptors (ACE2) in male, and also life style, such as higher levels of smoking and drinking among men as compared to women [32], this imbalance supports a higher susceptibility of men to develop severe respiratory diseases following COVID-19 infection. In addition, females with acute lung injury have higher rate of alveolar fluid clearance compared with men, which in turn lead to rapid resolution of pulmonary edema among female than male [33].

In this study, the odds of developing ARDS was nearly 4.5-fold higher among patients with oxygen saturation level <94% as compared to their counterparts. Similar finding reported from earlier studies [28, 34], in which poor oxygenation index was reported as cause and effect for ARDS development. The possible reason, poor oxygenation makes the lung stiff with positive end-expiratory pressure (PPEP). Circular relationship between oxygen level and ventilation depends on blood gas level which stemmed positive response to positive end-expiratory pressure (PEEP) is crucial in defining ARDS. Low oxygenation level by itself indicates severe respiratory failure; therefore, patients with low oxygen saturation level are more of at high risk to develop ARDS than their low-level peers.

### Limitation of the study

Since incomplete base-line data and undocumented outcome were excluded from the study, the recorded information might lack very important factors that could influence the prevalence of ARDS patients. on the other hand, this study did not evaluate treatment outcome. The nature of study can be biased different factors. Hence, generalization for others should be with caution.

## Conclusion

Prevalence of ARD among sever COVID-19 patients in the study area was significantly high. Factors like age65 years; low oxygen saturation and being male in gender were significantly associated. This implies that elders and patients with critical health conditions like (low oxygen saturation) have to get special attention in case of COVID-19 management to enhance good health outcome as well monitoring of the patient. Furthermore, strengthening the health care delivery system to satisfy the need of the patients should get due attention to reduce the prevalence of ARDS among COVID-19 patients.

## Supporting information

S1 Dataset (DTA)
